# Silver nanoparticles–decorated extracellular matrix graft: fabrication and tendon reconstruction performance

**DOI:** 10.1186/s40824-023-00428-0

**Published:** 2023-09-14

**Authors:** Sunfang Chen, Dan Cai, Qi Dong, Gaoxiang Ma, Chennan Xu, Xiaogang Bao, Wei Yuan, Bing Wu, Bin Fang

**Affiliations:** 1 Department of Orthopedics, the First Affiliated Hospital of Zhejiang Chinese Medical University (Zhejiang Provincial Hospital of Chinese Medicine), Hangzhou, 310000 China; 2 Department of Orthopedics, the Central Hospital Affiliated to Shaoxing University, Shaoxing, 312030 China; 3 Department of Orthopedics, the First People’s Hospital of Huzhou, First Affiliated Hospital of Huzhou University, Huzhou, 313000 China; 4 Department of Orthopedics, Honghui Hospital, Xi’an Jiao Tong University, Xi’an City, 710054 China; 5 Department of Orthopedics, The Spine Surgical Center, Second Affiliated Hospital of Naval Medical University, Shanghai, 200003 China; 6 Department of Orthopedics, Shanghai Municipal Hospital of Traditional Chinese Medicine, Shanghai University of Traditional Chinese Medicine, Shanghai, 200071 China

**Keywords:** Tendon graft, Extracellular matrix, Silver nanoparticles, Infection, Inflammation

## Abstract

**Background:**

The reconstruction of tendons with large defects requires grafts with high mechanical strength and is often hindered by complications such as infection and adhesion. Hence, grafts combining the advantages of mechanical resilience and antibacterial/antiadhesion activity are highly sought after.

**Methods:**

The silver nanoparticles (GA-Ag NPs) synthesized from gallic acid and silver nitrate were attached to a decellularized extracellular matrix (Decellularized Tendon crosslinking GA-AgNPs, DT-Ag). We examined the histological structure, mechanical property, morphology, Zeta potential, cytotoxicity, antibacterial properties, antioxidant and anti-inflammatory properties, and ability of the DT-Ag to treat tendon defects in animals.

**Results:**

Approximately 108.57 ± 0.94 μg GA-Ag NPs loaded per 50 mg DT, the cross-linked part of GA-Ag NPs was 65.47 ± 0.57%, which provided DT-Ag with long-lasting antibacterial activity. Meanwhile, GA endowed DT-Ag with good antioxidant and anti-inflammatory activities. Additionally, The DT-Ag facilitated M2 macrophage polarization, and suppressed fibrin deposition by hindering fibroblast adhesion. Mormore, the main advantages of DT-Ag, namely its long-lasting antibacterial activity (tested using *Escherichia coli* and *Staphylococcus aureus* as models) and the ability to prevent tissue adhesion were confirmed in vivo.

**Conclusion:**

The fabricated multifunctional tendon graft was highly hydrophilic, biocompatible, and mechanically resilient, and concluded to be well suited for dealing with the main complications of surgical tendon reconstruction and has bright application prospects.

**Graphical Abstract:**

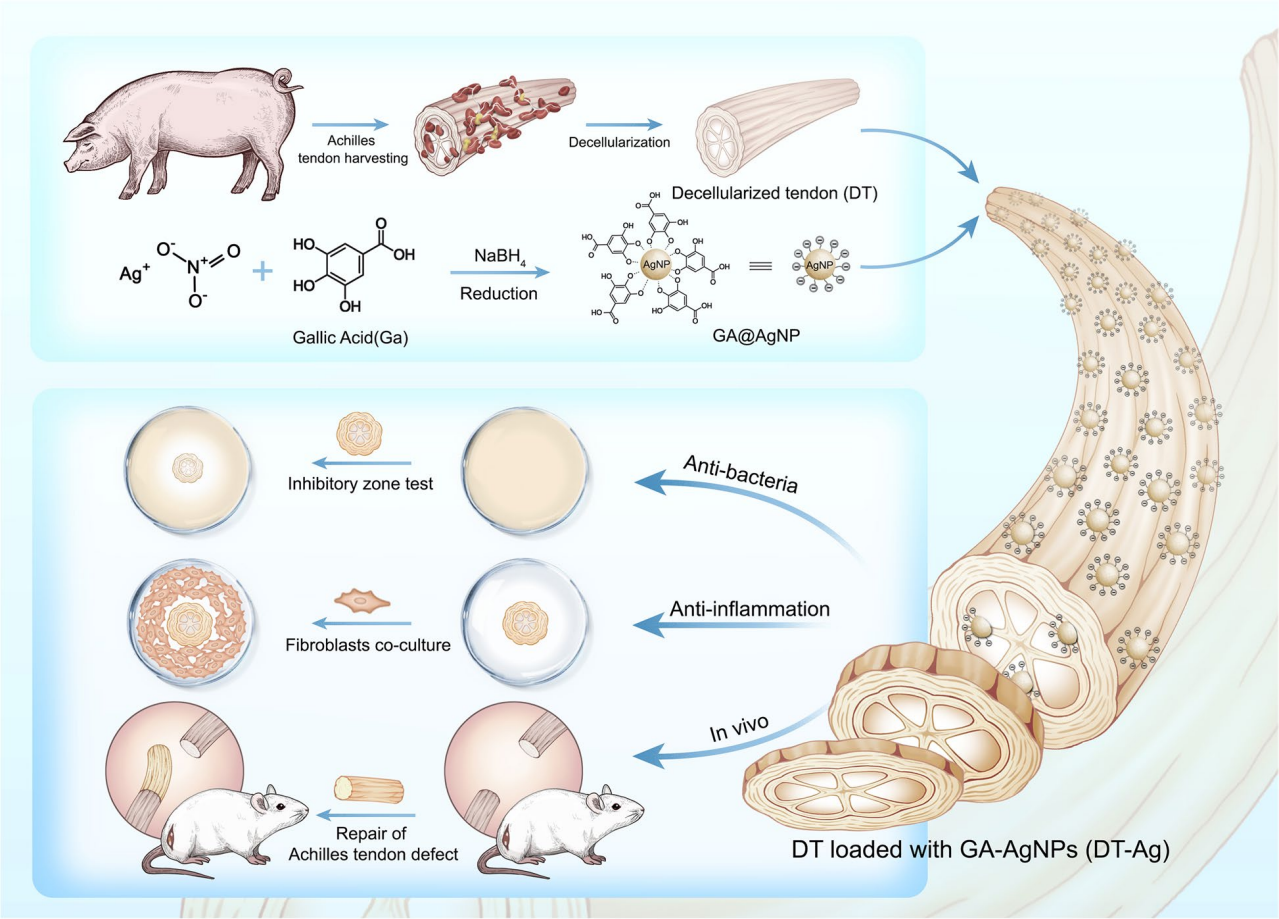

**Supplementary Information:**

The online version contains supplementary material available at 10.1186/s40824-023-00428-0.

## Introduction

The reconstruction of ruptured tendons is surgically challenging because of the substantial gap created by the retraction of their ends and poor self-heal ability due to the low blood vessel supply. Although small defects can be closed using simple sutures [1], large ones often require strong mechanically fixed grafts [2]. According to their source, transplanted tendons are mainly classified as autologous, allogeneic, and synthetic [3]. Because of the shortage of autologous tendons, synthetic and allogeneic ones have become the most widely used transplantation substitutes. Tendon reconstruction involving grafts is often hindered by postoperative tissue adhesion and infection [4], both of which may lead to the complete loss of tendon functions. In particular, tissue adhesion is caused by inflammation and fibrous protein deposition [5] and starts in the inflammatory stage after tendon injury. In this stage, inflammatory parasecretory cells recruit inflammatory cells to elicit an immune response, while the adhesion of fibroblasts leads to fibrin deposition. The severe inflammatory reaction consumes immune cells [6] and weakens the autologous anti-infection activity to facilitate infection. Moreover, mechanical injury, infection, and foreign body reactions during invasive surgery can promote the excessive production of reactive oxygen species, which induce oxidative stress and promote the release of inflammatory factors. In turn, the increased inflammation aggravates oxidative stress and eventually causes coagulation and fibrin formation, thus directly accelerating adhesion [7]. Therefore, the above problems can ideally be solved through the development of tendon grafts with antibacterial and antiadhesion activities.

Nowadays, overuse of antibiotics has lead to multiple drug-resistant bacteria, biofilms and intracellular infections. As a result, the failure of traditional antibiotics has given rise to many new types of nanotherapy [8–11]. Nanoparticle-mediated therapeutics have emerged as potential candidates for antibacterial treatment due to their suitable dimensions, penetration capacity, and high efficiency in targeted drug delivery[10]. Silver nanoparticles (Ag NPs) exhibit antibacterial, anti-inflammatory, and wound-healing effects and can therefore potentially find clinical applications [12]. The strong ability of Ag NPs to inhibit the proliferation of a broad range of bacteria and fungi is due to the continuous release of Ag^+^ ions, which can destroy bacterial membranes and disrupt the functions of cellular enzymes and other proteins [13]. However, the ability to penetrate cell membranes also makes Ag NPs toxic to human cells [14]. Given that cytotoxicity of Ag NPs is concentration-dependent, it can be reduced by loading these NPs into biological materials, e.g., through in situ formation [15] or deposition [16], to ensure slow release. Ag NPs also inhibit fibroblast differentiation [17], promote tendon repair [18] and M2 macrophage polarization [19]. Studies on the prevention of adhesion after tendon surgery often use Ag NPs as healing-promoting and bactericidal implant components, whereas studies on the direct antiadhesion activity of Ag NPs are few. Implant hydrophilization is known to hinder adhesion [20, 21]. Moreover, Verma et al. [22] and Rosso et al. [23] found that some materials with high negative charge can prevent the adhesion and migration of fibroblasts. Therefore, postoperative adhesion can potentially be hindered by endowing the Ag NPs to be loaded into implants with a negative charge such as gallic acid molecule.

To date, scaffold materials have been applied to tissue repair/reconstruction [24]. Autologous, allogeneic, and synthetic grafts are commonly used for tendon healing but can easily cause structural and functional dysfunction at the tendon site and are inapplicable to large defect sites because of the limited sources. Autologous tendon transplantation is the gold standard for patients with small defects, while allograft tendon transplantation can lead to rejection, infection, and other complications. Synthetic grafts such as those constructed from polyethylene terephthalate, polylactic acid, or poly [D, L-lactic-co-glycolic acid] [24, 25] may elicit rejection in vivo via a currently unclear mechanism [26] and afford decomposition products with certain toxicity [27]. Compared with synthetic polymer tendons, decellularized allogeneic tendons have highly homologous structures and are much less likely to induce rejection because of their natural origin. Moreover, decellularized tendons have the same perfect spatial structure as normal tendons and retain some factors (in the extracellular matrix) capable of inducing the growth and differentiation of myosatellite cells, which can greatly promote the regeneration of homologous tissues [28]. Tao et al. studied animal tendon repair, showing that the use of decellularized tendons can not only prevent tendon adhesion but also achieve higher tendon repair quality [29]. Therefore, the combination of antimicrobial Ag NPs and decellularized allogeneic tendons may help to solve the problems of tendon reconstruction.

**Scheme1:**
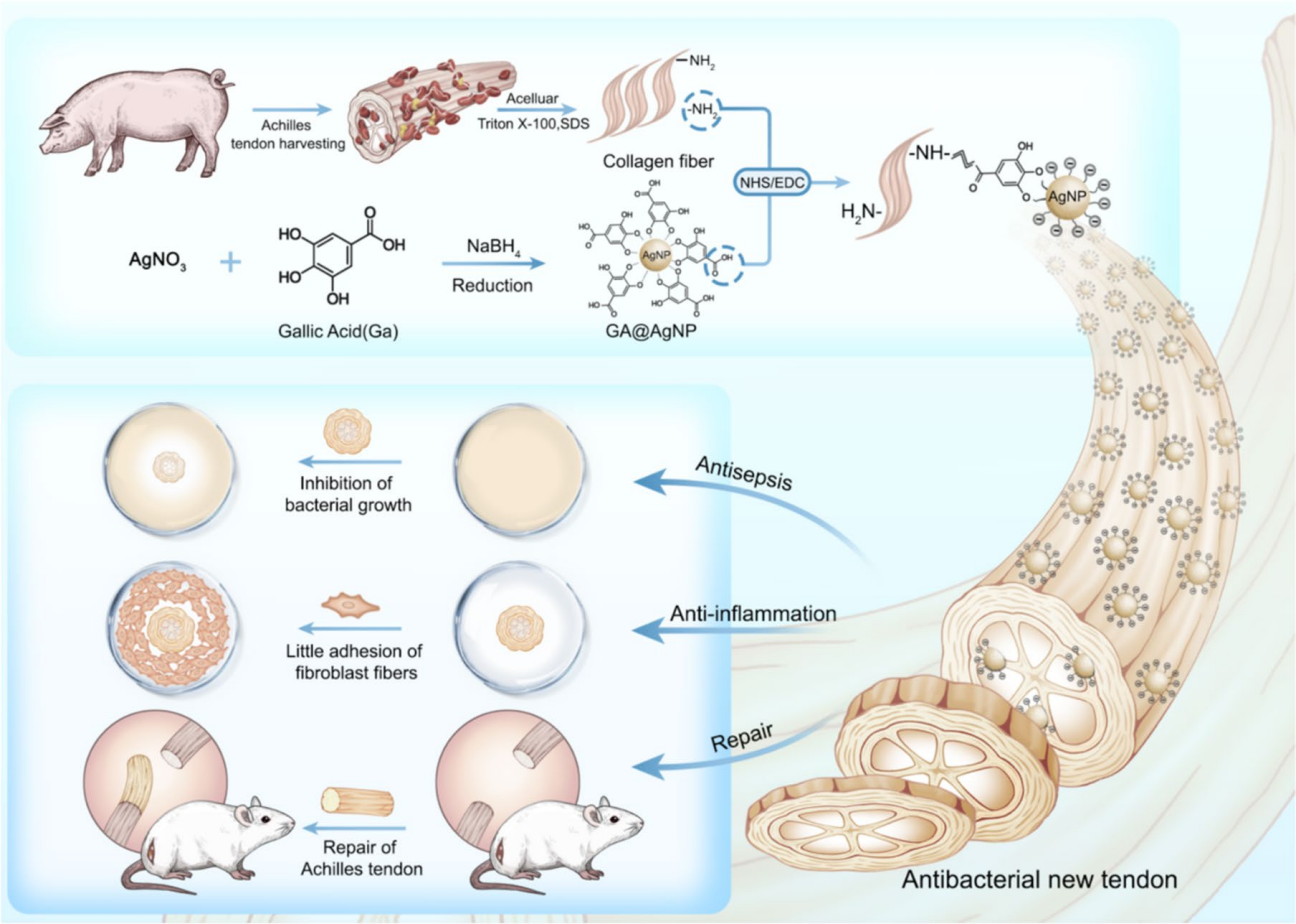
The schematically illustration the structure of the GA-Ag-NPs, and subsequent the GA-Ag-NPs-attached decellularized ECM for tendon reconstruction

Inflammation is an inevitable early immune response in natural wound healing and plays a crucial role in tissue regeneration. Macrophages are one of the most important effector cells involved in substance-induced inflammatory responses [30]. Many studies indicate that the M1 to M2 macrophage transformation must be temporarily manipulated to prevent excessive inflammation from damaging normal tissue and delaying wound healing [31, 32]. The decellularized extracellular matrix (ECM) has a good chemotactic ability to induce macrophage polarization [32], while Ag NPs can induce M2 macrophage polarization. Considering the above, we herein prepared negatively charged gallic acid (GA)-Ag NP composites (GA-Ag NPs) by reducing silver nitrate with gallic acid (GA) molecule. Subsequently, the carboxyl groups exposed on GA-Ag-NPs can chemically cross-linked with the amino groups exposed on the decellularized ECM through EDC/NHS to form the GA-Ag-NPs-attached decellularized ECM scaffold. GA can endow anti-inflammatory and anti-adhesion properties, while Ag nanoparticles confer antibacterial capability (Scheme 1). The results of tissue staining, DNA content, collagen content, cytotoxicity, mechanical property, and scanning electron microscopy (SEM) analyses demonstrated that the fabricated graft also possesses good biocompatibility and mechanical properties.

## Experimental section

### Preparation and characterization of GA-Ag NPs

GA-Ag NPs were prepared using a slight modification of a previously reported method [33], with specific moles such as silver nitrate (1 mol), gallic acid solution (1 mol), and NaBH4 (6 mol). An aqueous solution containing GA and silver nitrate was dropwise added to a 4 °C precooled alkaline solution of sodium borohydride using an infusion pump at a rate of 80 drops per hour under the condition of light protection, and the mixture was magnetically stirred for 2 h. The solution was agitated using a constant-temperature shaker at 25 °C and 200 rpm for 5 h, centrifuged at 15,000 rpm, and the precipitate was redispersed in deionized water for further use.

### Antibacterial activity of GA-Ag NPs

Two typical bacterial strains (*S. aureus* and *E. coli*) were used for antibacterial activity evaluation. The bacteria were added to sterile test tubes containing 10 mL of the Luria–Bertani broth and cultured overnight at 37 °C. The bacterial suspension (10^6^ CFU/mL) was evenly applied on the agar plate three times using a sterile cotton swab. Then, a sterile blank drug-sensitive paper was impregnated with 10 μL of the tested GA-Ag NP solution, dried, and placed on the seeded agar plate. After 18-h incubation at 37 °C, the diameter *D* of the inhibition zone (mm) was calculated as *D* = outside diameter – inner diameter. The outer diameter refers to the size of the antibacterial zone, and the inner diameter refers to the blank drug-sensitive paper.

### Preparation and characterization of the decellularized tendon (DT)

Fresh tendons were obtained from local slaughterhouses. The native tendon (NT) was dissected and rinsed with sterile water three times for three consecutive meltings (− 80 to 37 °C) cycles. Tissues were soaked with 1vol% Triton X-100 solution for 6 h and 0.1% sodium dodecyl sulfonate for 24 h at 120 rpm and constant temperature (25 °C) and rinsed with PBS solution for 12 h between each step. The obtained DT was washed with phosphate-buffered saline (PBS) several times and sterilized by γ-irradiation at a dose of 25 kGy.

NT and DT were pruned to an appropriate size and stained with hematoxylin and eosin (H&E) to observe the tissue structure or with 4',6-diaminyl-2-phenylindole (DAPI) to observe residual nuclei. DNA was extracted using the DNeasy Blood & Tissue Kit according to the manufacturer’s specifications, and residual DNA in the tissue was quantified using the NanoDrop8000 spectrophotometer. The hydroxyproline assay kit was used for collagen quantitation. NT and DT morphologies were examined using SEM (JSM-6360LV, JEOL, Japan). Before imaging, the sample was sputtered with gold for 60 s and an accelerated voltage of 5–15 kV was used.

For mechanical property testing, the samples were tensioned using an Instron tension system, straightened and adjusted using a 0.1-N preload, and loaded at a rate of 10 mm/min until failure. The failure mode was recorded, and the failure load (N) was obtained from the load–displacement curve. The failure load was normalized with respect to the sample cross-sectional area to calculate the ultimate stress (MPa). During the test, a 0.9% saline solution was applied to prevent sample dehydration.

### Preparation of DT decorated with GA-Ag NPs (DT-Ag)

DT was immersed into a solution of GA-Ag NPs with a certain concentration, soaked for 30 min under continuous agitation, thoroughly rinsed with deionized water to remove physically adsorbed GA-Ag NPs, placed in an EDC/NHS solution (EDC = 2NHS = 40 mM), magnetically stirred at room temperature for 6 h or overnight, rinsed with deionized water, and dried to afford DT-Ag. Lyophilized DT and DT-Ag were partly processed into 1 cm × 1 cm thin slices and partly ground into powders for subsequent study.

### Loading and attachment efficiency of GA-Ag NPs

A linear concentration-absorbance relationship was established based on the results of ICP-OES analysis and a microplate reader (Multiskan Sky, America) to quantify GA-Ag NP content. DT (50 mg) was immersed into a GA-Ag NP solution (300 μL; Ag NP mass = *m*_0_). After soaking for 30 min, the excess GA-Ag NP solution (Ag NP mass = *m*_1_) was removed. The mass of loaded GA-Ag NPs $({M}_{\mathrm{load}})$ and loading efficiency ${\chi }_{\mathrm{load}}$ was calculated as

$${M}_{\mathrm{load}}= {m}_{0} - {m}_{1},$$

$${\chi }_{\mathrm{load}}=({m}_{0}-{m}_{1})/{m}_{0}.$$

The EDC concentration (40 mM) and the EDC/NHS molar ratio (2:1) were selected according to a previous report [34]. After the attachment of GA-Ag NPs (150 rpm, 6 h), DT-Ag was washed in DDH_2_O (double steaming water) several times. For attachment efficiency (*χ*_attachment_) determination, samples were subjected to several cycles of ultrasonication-assisted washing. When the amounts of residual GA-Ag NPs in DT (*m*_2_) and DT-Ag (*m*_3_) stopped changing, they were recorded, and *χ*_attachment_ was calculated as

$${\chi }_{\mathrm{attachment}}=100\mathrm{\% }\times ({m}_{3}-{m}_{2})/{M}_{\mathrm{load}}.$$

### In vitro release of GA-Ag NPs

The release of GA-Ag NPs from DT-Ag was analyzed using a microplate reader. Freeze-dried DT-Ag (50 mg) was soaked in PBS buffer (10 mL, pH = 7.2) and agitated by shaking at room temperature and 150 rpm. The PBS solution was removed at a preset time point and replaced with the same amount of fresh PBS. The collected liquid was filtered through a 0.22-μm filter and analyzed using the microplate reader.

To further evaluate the release of GA-Ag NPs after their attachment, we treated the samples using an ultrasonic processor (Thermo, T-701FB120220) and collected and quantified the released GA-Ag NPs as above. Ultrasonic treatment consisted of 30-s ultrasonication at an amplitude of 50% followed by 30-s rest. An aliquot of the liquid was imaged by TEM to detect scattered Ag NPs.

### Antibacterial activity evaluation

Inhibition zone tests were performed according to the AATCC 90–2011 method for *E. coli* (ATCC25922) and *S. aureus* (ATCC6538) as model bacteria [35]. Several Petri dishes containing the sterile nutrient (agar; 15 mL) were prepared for cooling. The required bacterial solution (1 × 10^6^ CFU/mL) was taken up using a sterile cotton swab and evenly smeared on a Petri dish. The smearing procedure was repeated three times (a new cotton swab was used each time). DT or DT-Ag trimmed to a diameter of 8 mm were placed in the center of the inoculated Petri dish and incubated at 37 °C for 14 h. The Petri dishes were photographed, and *D* was determined as mentioned above.

For further antibacterial activity analysis, DT or DT-Ag (500 mg) was washed several times with sterile water, cut into small pieces, and put into a tube containing PBS (10 mL) with a bacterial concentration of 1 × 10^4^ CFU/mL. The tube was incubated at 37 °C upon shaking at 150 rpm. At a predetermined time, a 20-μL aliquot of the bacterial solution was sampled and spread on a Petri dish. The dish was incubated at 37 °C for 18 h, and colony-forming units were counted. The samples were then placed in 10% paraformaldehyde and fixed for 4 h. A part of the DT was cut into 5-μm-thick slices (Leica CM1950) and stained with PI. The other part was dehydrated using an ethanol gradient, sputter-coated with Au–Pd, and observed by SEM.

### Hemolysis test

The hemolysis test was performed as described by Tao et al. [29]. Rat blood (10 mL) was gently stirred to remove fibrin and supplemented with normal saline (10 mL). After thorough mixing, the solution was centrifuged at 2000 rpm for 5 min, and the supernatant was discarded. The above steps were repeated 3–5 times until the supernatant became colorless and transparent. The thus obtained erythrocytes were suspended in normal saline at a content of 2 vol%, and each of the four centrifuge tubes was sequentially supplemented with this suspension (0.5 mL), normal saline (1 mL), DT extract (1 mL), DT-Ag extract (1 mL), or distilled water (1 mL). The tube contents were thoroughly mixed, incubated at 37 °C (water bath) for 3 h, and centrifuged at 2000 rpm for 5 min. The supernatant from each tube (200 μL) was collected, and its absorbance at 450 nm was measured using a microplate reader.

### Antioxidant and anti-inflammatory activities

The 2,2-diphenyl-1-picrylhydrazyl (DPPH) radical scavenging assay was used to determine the free radical scavenging abilities of DT and DT-Ag [36]. GA solutions with various concentrations, DT, and DT-Ag prepared using different GA-Ag NP concentrations were placed in ethanolic DPPH solution (1 mL, 0.2 mM). The mixtures were kept in the dark for 30 min, and their absorbances were then recorded at 517 nm. DDH2O was used as a negative control (NC), and ascorbic acid solutions with different concentrations were used as positive controls. The antioxidant activity (inhibition rate) was calculated from NC and sample absorbances (*A*) as

$$\mathrm{Inhibition rate }(\mathrm{\%})=100 \times ({A}_{\mathrm{NC}}-{A}_{\mathrm{sample}})/{A}_{\mathrm{NC}}.$$

As described by Sharifi-Rad et al. [37], we used a slightly modified human erythrocyte membrane stability assay to determine anti-inflammatory activity. Rat blood samples were washed with 0.85% NaCl solution (pH 7.2) and prepared into a 10 vol% cell suspension. Low saline solution (2 mL, 0.36%), pH 7.4 phosphate buffer (1 mL, 0.15 M), and rat erythrocyte suspension (0.5 mL) were mixed, and the mixture was treated with the sample of choice, incubated at 37 °C for 30 min, and then centrifuged at 4000 rpm for 15 min. The supernatant was collected, and its hemoglobin content was determined from its absorption at 560 nm. DDH_2_O was used as a NC, and diclofenac (anti-inflammatory drug) at different concentrations was used as a positive control. The protection (%) of erythrocyte membrane stability was determined from NC and sample absorbances (*A*) as

$$\mathrm{Protection }(\mathrm{\%})=100 \times ({A}_{\mathrm{NC}}-{A}_{\mathrm{sample}})/{A}_{\mathrm{NC}}.$$

## Macrophage polarization analyse

### Immunofluorescence method

M1 (iNOS) and M2 marker (CD206)-expressing macrophages were analyzed by an immunofluorescence method. RAW 264.7 cells were cocultured with scaffold (1 × 10^5^ cells/well) for 1 day. The macrophages were then scraped off and seeded on a new plate for reattachment, which was followed by fixation with paraformaldehyde/PBS (4 vol%), permeabilization with Triton X-100, and closure with 5% normal goat serum for 1 h. Next, the specimens were incubated with primary antibodies (ab210823 and ab64693) overnight at 4 °C. Secondary antibodies (ab6785 and ab6939) were incubated with the sample for 1 h. After restaining with DAPI for 10 min, the cells were detected by confocal laser scanning microscopy (CLSM, TCS SP2; Leica, Wetzlar, Germany). Lipopolysaccharide (LPS) induction (200 ng/mL) was used as the M1 positive control, while IL-4 induction (20 ng/mL) was used as the M2 positive control.

### Reverse transcription-quantitative polymerase chain reaction (RT-qPCR)

RAW 264.7 cells were inoculated on the sample at a density of 1 × 10^5^ cells/well. After 1-day incubation, RNA was collected using a Yew kit, and reverse transcription was performed using a reverse transcription kit (Yew) according to manufacturer's instructions. The expression levels of the housekeeping gene GADPH were used to normalize those of the genes of interest. The primers used for the real-time qPCR are listed in Table S1.

### In vivo experiment

Twenty-four SD (sprague–dawley) rats (male, three months old, 400–450 g) were randomly divided into autologous tendon (AT, *n* = 8), DT (*n* = 8) and DT-Ag (*n* = 8) groups. They were anesthetized with ketamine (30.0 mg/kg). The tendon was exposed through a midline incision of the skin, subcutaneous tissue, and fascia 3 mm from the distal gastrocnemius and 3 mm above the calcaneus. Immediately after the tendon incision, the defect was filled with a 6-mm AT, DT, or DT-Ag, and a suspension of *S. aureus* was injected (100 μL, 1 × 10^4^ CFU/mL). A 6–0 PDS absorbable suture was used to close the tendon, and a 3–0 nonabsorbable suture was used to close the skin. All procedures were performed under sterile conditions, and the rats were allowed to move freely in their cages after surgery.

### Adhesion scoring

Three weeks after the operation, the adhesion around the tendon was evaluated and scored by two observers. The specific scoring criteria [29] were as follows: no adhesion = 0; slight adhesion, blunt separation = 1; moderate adhesion, not easy to separate = 2; severe adhesion, unable to separate = 3.

### Blood test

To evaluate the possible side effects of surgical treatment, we performed blood tests one week after surgery and assessed the complete blood count, renal function (creatinine and uric acid contents), and liver function (alanine aminotransferase and aspartate aminotransferase activities).

### Histological evaluation

After three weeks, the rats were euthanized, and the tendons were removed for histological analysis. H&E staining was performed to observe tendon growth and healing. The anticd68 primary antibody was used to locate macrophages, and myeloperoxidase (MPO) was used to locate neutrophils.

## Results

### Preparation and characterization of GA-Ag NPs

**Fig. 1:**
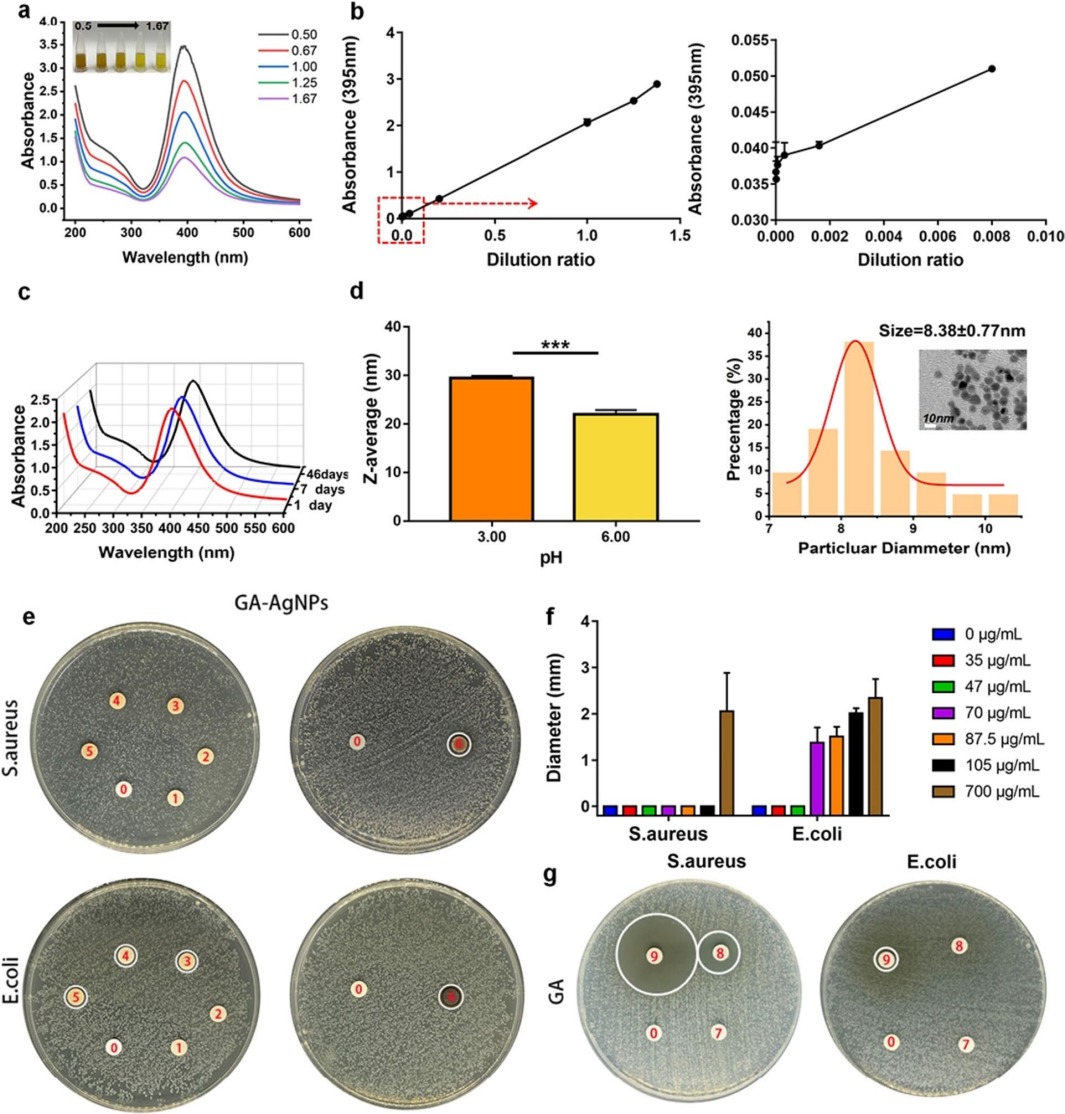
**a** Absorption spectra of GA-Ag NP solutions with different concentrations. **b** Absorbance–dilution ratio curve of GA-Ag NP solution. **c** Absorbance change of GA-Ag NP solution over 46 days. **d** Particle size of GA-Ag NPs as a function of pH and related TEM images. **e**, **f** Setup and results of the inhibition zone test performed for GA-Ag NPs, with numbers 0 to 6 representing concentrations of 0, 35, 47, 70, 87.5, 105, and 700 μg/mL, respectively. **g** Results of the inhibition zone test performed for GA, with numbers 7 to 9 representing concentrations of 700, 7000, and 70,000 μg/mL, respectively. ***: *p* < 0.001

The stabilizing action of GA, i.e., its ability to prevent the aggregation of GA-Ag NPs and facilitate their dispersion, was ascribed to its hydrophilic carboxyl group [33]. The UV absorption spectra of GA-Ag NPs exhibited a characteristic surface plasmon resonance peak near 395 nm (Fig. 1a). With decreasing concentration, the color of the GA-Ag NP solution became less intense, and absorption decreased (Fig. 1b). The absence of significant absorption peak change after 46-day storage (Fig. 1c) indicated excellent stability. Our previous studies demonstrated that infection induces a slight change in the pH of the internal environment, and the pH measured by blood gas analysis was 6.1 ± 0.6 [38]. The absorption peak of GA-Ag NPs disappeared at pH < 3 (Fig. S1a), possibly because of the protonation of GA under acidic conditions and the resulting agglomeration of GA-Ag NPs. This behavior was similar to previously reported results [33]. However, at pH 4.0–11.00, the shape of the absorption peak did not significantly change. The mean particle size significantly (*p* < 0.001, Fig. 1d) changed upon going from pH 3.0 (29.47 ± 0.43 nm) to 6.0 (22.01 ± 0.87 nm) (Fig. 1d). Furthermore, GA-Ag NPs existed as well-formed well-dispersed spherical nanoparticles at pH 6.0 (Fig. 1d) but experienced slight agglomeration at pH 3.0 (Fig. S1b). Based on the results of TEM imaging, the average diameter at pH 6.0 was determined as 8.38 ± 0.77 nm (Fig. 1d). This suggests the possible formation of silver nanoparticles with putaminal structure.

### Antibacterial activity of GA-Ag NPs

**Fig. 2:**
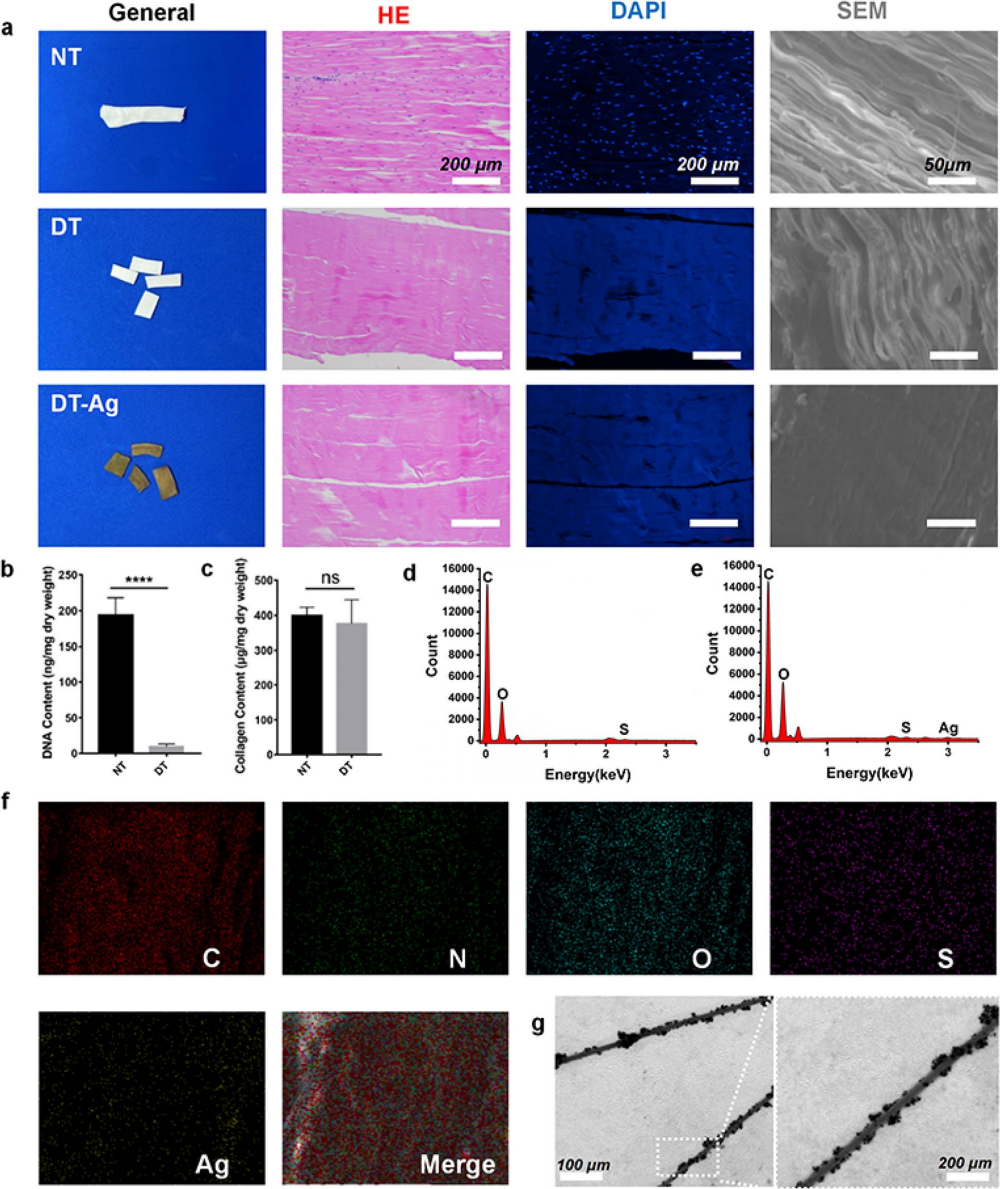
**a** Macroscopic, H&E, DAPI, and SEM images of NT, DT, and DT-Ag. **b** DNA contents of NT and DT. **c** Collagen retention behaviors of NT and DT. **d**, **e** Results of EDS analysis for DT and DT-Ag. **f** Results of EDS analysis and element distribution diagram obtained for DT-Ag. **g** TEM image showing GA-Ag NPs attached to the fibers of DT-Ag

**Fig. 3:**
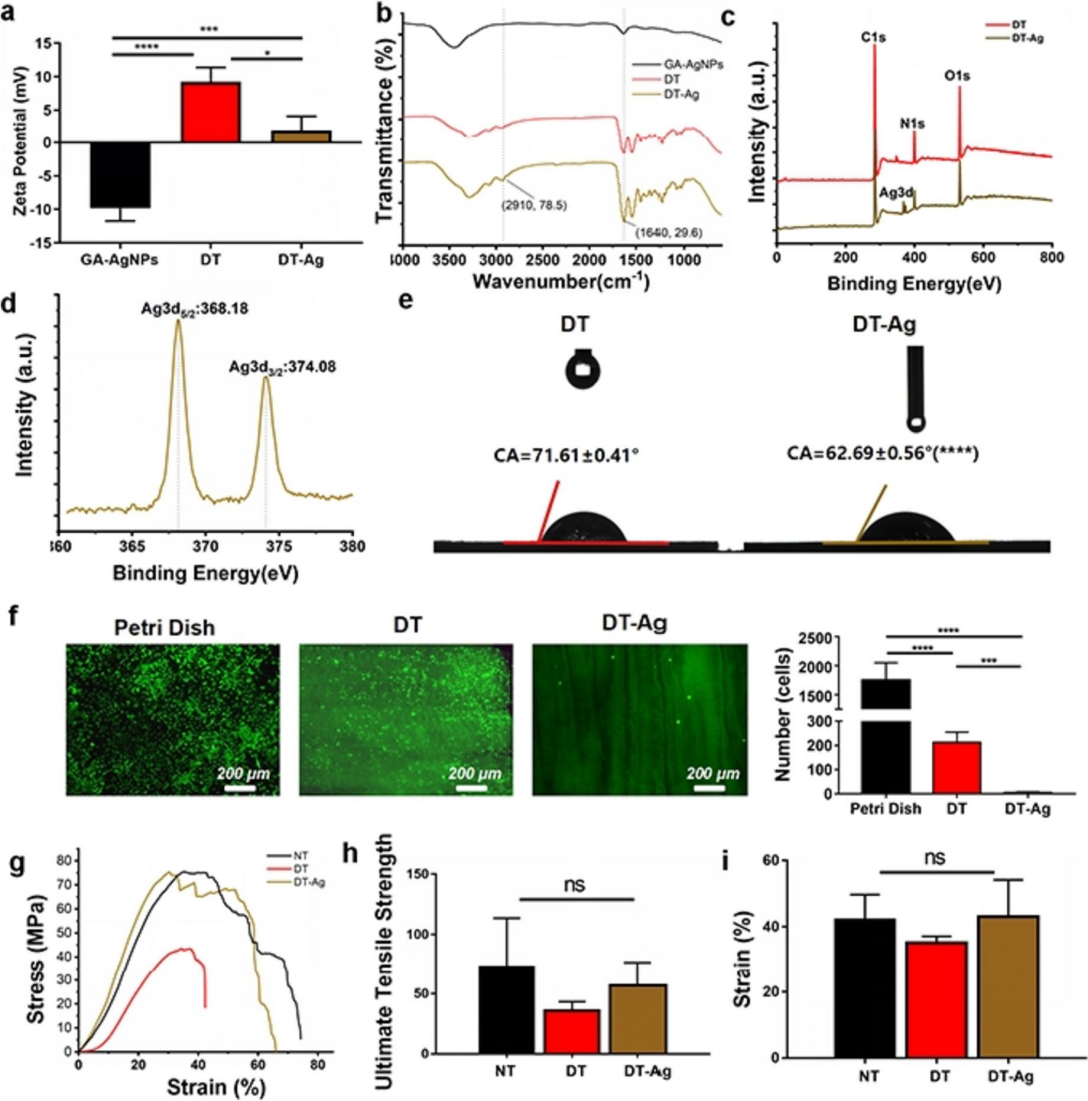
**a** Zeta potentials of GA-Ag NPs, DT, and DT-Ag. **b** Infrared spectra of GA-Ag NPs, DT, and DT-Ag. **c** Survey X-ray photoelectron spectra of DT and DT-Ag. **d** Ag 3d spectrum of DT-Ag. **e** Water contact angles of DT and DT-Ag. **f** Calcein staining images of fibroblast growth on culture dish, DT, and DT-Ag surfaces. **g**–**i** Stress–strain curves, limit stresses, and failure strains of NT, DT, and DT-Ag

**Fig. 4:**
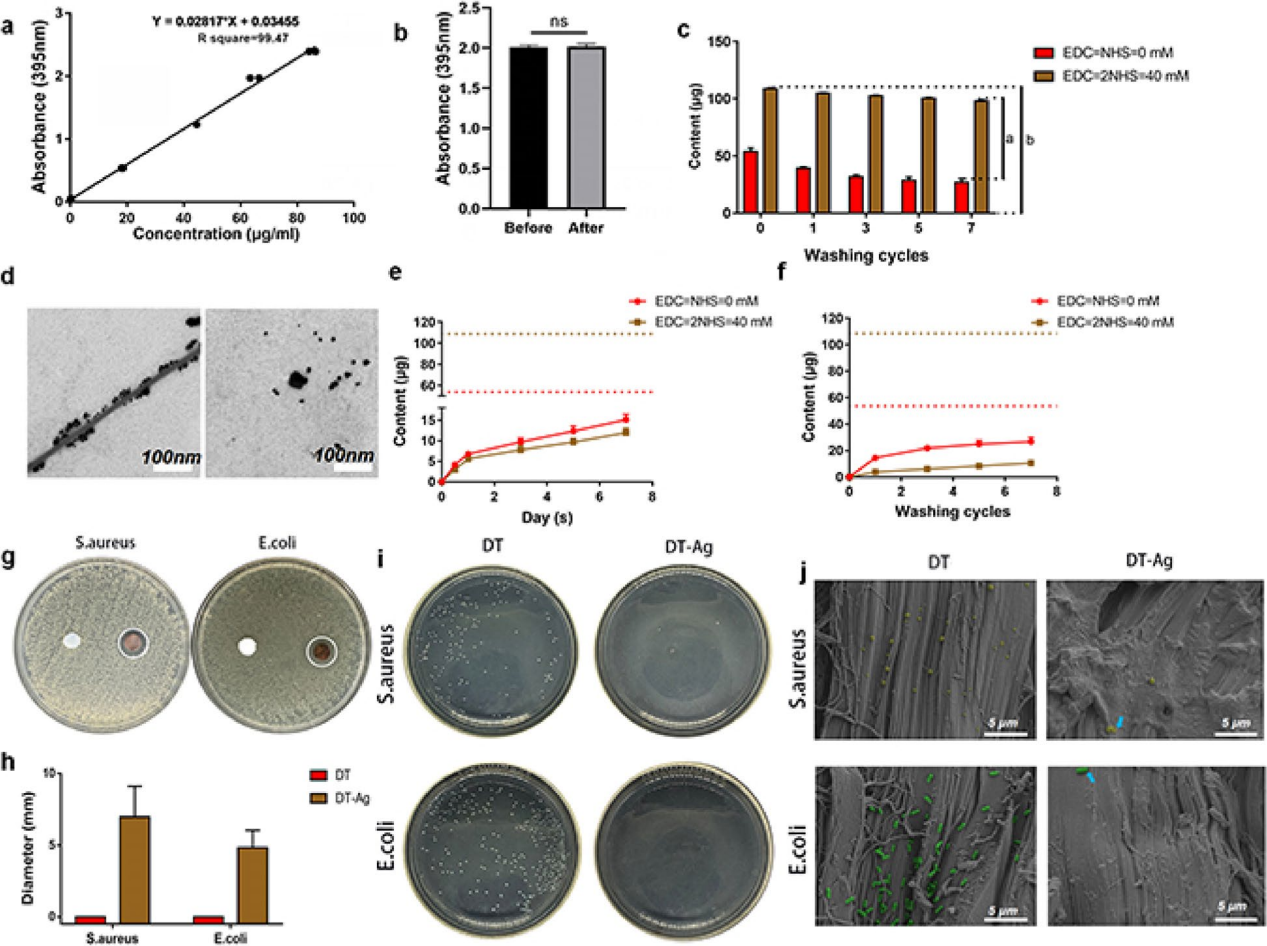
**a** Concentration–absorption curve of GA-Ag NP solution. **b** Change in nanosilver concentration due to DT soaking in GA-Ag NP solution. **c** Residual amounts of Ag NPs on the tendons of the nongrafted ([EDC] = [NHS] = 0; DT) and grafted ([EDC] = [2NHS] = 40 mM, DT-Ag) groups after different numbers of ultrasonication cycles, where “*a*” is the relative GA-Ag NP grafting amount, “*b*” is the total amount of GA-Ag NPs, and *χ*_attachment_ can be calculated by *a*/*b* × 100%. **d** Left: TEM image showing fibers and GA-Ag NPs in the grafted group after ultrasonication; right: TEM image showing GA-Ag NPs scattered in the solution. **e** Continuous release of GA-Ag NPs in nongrafted and grafted groups assessed using a shaking table. **f** Continuous release of GA-Ag NPs in the two groups assessed after different times of ultrasonic cleaning. **g**, **h** Results of inhibition zone tests obtained for DT and DT-Ag thin slices and related statistical analysis. **i** Activities of DT and DT-Ag against planktonic bacteria. **j** SEM images of DT and DT-Ag after incubation with bacteria

**Fig. 5:**
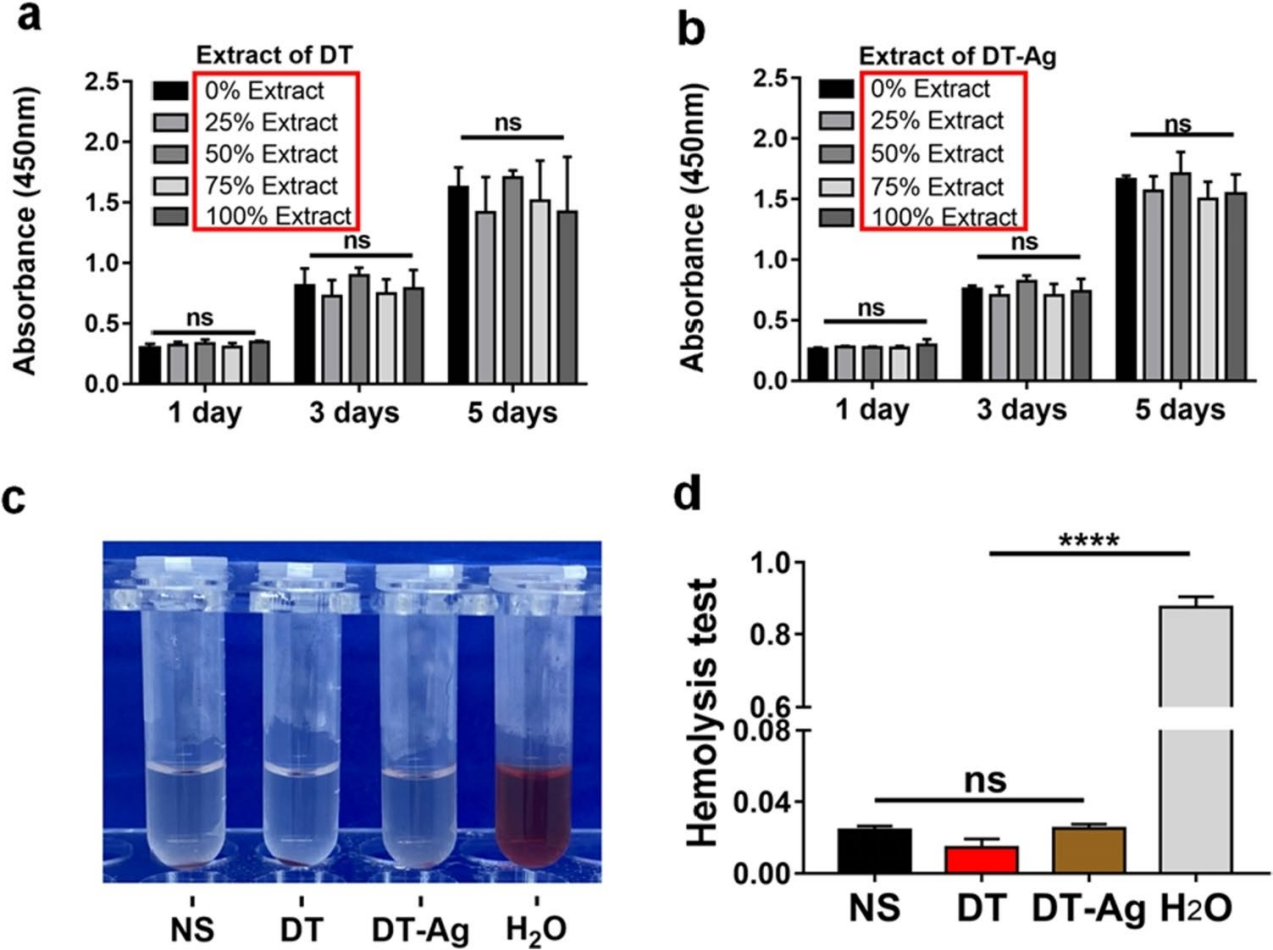
Cytotoxicity test results obtained for (**a**) DT and (**b**) DT-Ag. Hemolysis test results of all samples. Normal saline and water were used as negative and positive controls, respectively. **c** is the qualitative test, while (**d**) is the quantitative test. ****: *p* < 0.0001

**Fig. 6:**
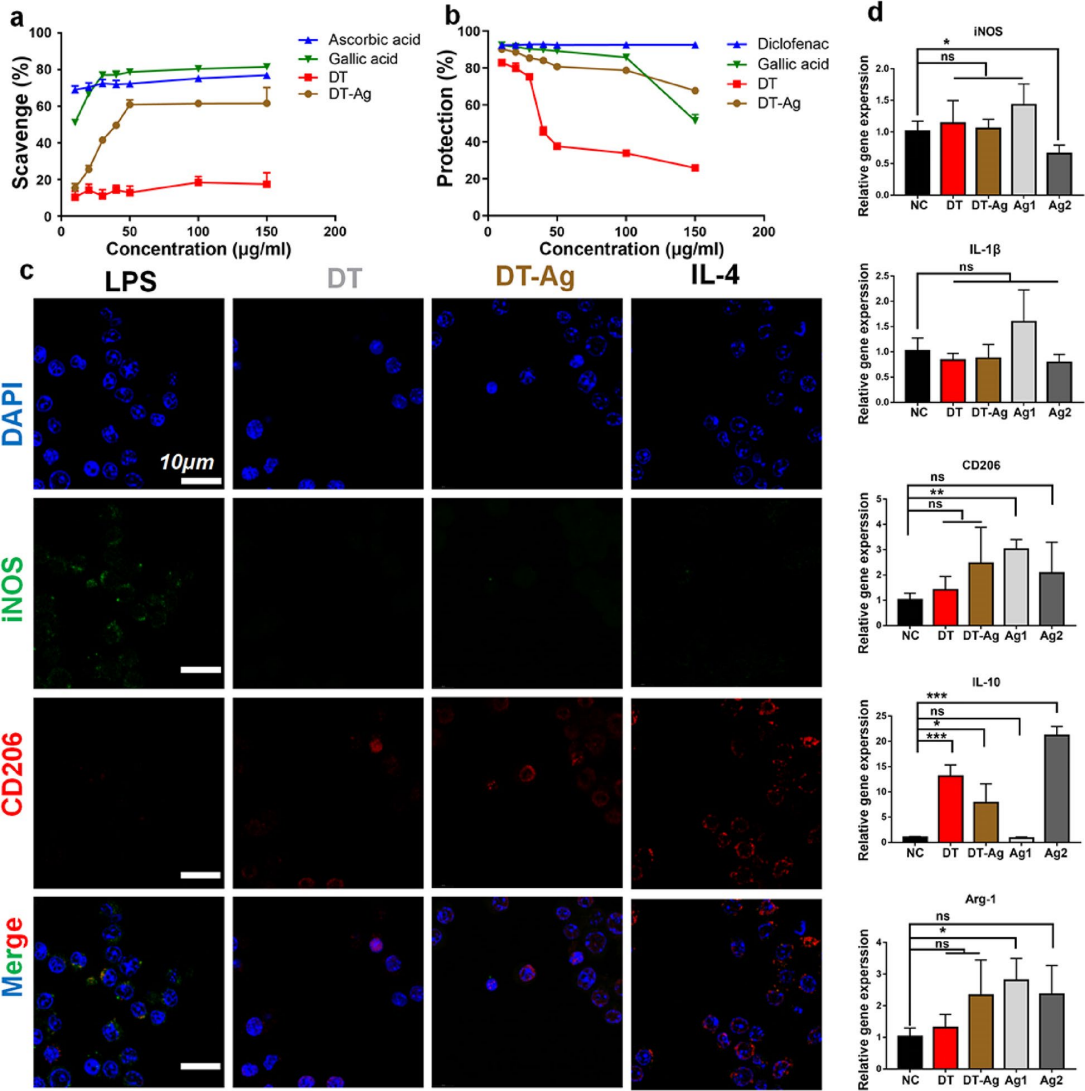
**a** Free radical scavenging abilities of GA, DT, and DT-Ag in the concentration range of 10–150 μg/mL. Ascorbic acid was used as a positive control. **b** Anti-inflammatory activities of GA, DT, and DT-Ag in the concentration range of 10–150 μg/mL. Diclofenac was used as a positive control. **c** Results of the immunofluorescence analysis of RAW 264.7 macrophages treated with LPS (200 ng/mL), DT, DT-Ag, and IL-4 (20 ng/mL) for 24 h. **d** Expression levels of M1- (iNOS and IL-1b) and M2-type markers (CD206, Arg-1, and IL-10) in RAW 264.7 macrophages after 24-h coculturing with NC, DT, DT-Ag, Ag1, and Ag2. NC: Negative control with only DMEM added; Ag1: 5 μg/mL GA-Ag NPs; Ag2: 10 μg/mL GA-Ag NPs. *: *p* < 0.05, **: *p* < 0.01, ***: *p* < 0.001

At low concentrations of GA-Ag NPs (35–105 μg/mL), the *S. aureus* inhibitory zones were difficult to detect (Figs. 1e - 5), whereas, at a GA-Ag NP concentration of 700 μg/mL, the inhibition zone was well visible (*D* = 2.06 ± 0.83 mm; Figs. 1e, 6). As in the case of *E. coli*, the size of the inhibitory zone increased with increasing GA-Ag NP concentration (Fig. 1e and f). The *S. aureus* inhibitory zone had a diameter of 2.06 ± 0.83 mm at a GA-Ag NP concentration of 700 μg/mL, while values of 1.37 ± 0.34, 1.51 ± 0.22, 2.01 ± 0.11, and 2.35 ± 0.41 mm were observed for *E. coli* at GA-Ag NP concentrations of 70, 87.5, 105, and 700 μg/mL, respectively. In cases other than those presented above, no obvious inhibitory zone was observed. Figure 1f more directly visualizes the antibacterial activity of GA-Ag NPs, indicating that both high and low Ga-Ag NP concentrations were more lethal to *E. coli* than to *S. aureus*, while both bacteria were effectively inhibited at high concentrations. The bacteriostatic activity of pure GA was also analyzed (Fig. 1g, Fig. S2a), but no obvious bacteriostatic zone was observed at 700 μg/mL. At 7000 μg/mL, GA exhibited a certain activity against *S. aureus* but not against *E. coli*, and a large bacteriostatic zone was observed for both bacteria at 70,000 μg/mL. Thus, compared to GA, GA-Ag NPs featured a higher activity against both *E. coli* and *S. aureus*, particularly against *E. coli*.

### Preparation and characterization of DT-Ag

Figure 2a presents images of stained NT, DT, and DT-Ag. The images of H&E-stained samples demonstrated that most nuclei were removed, while the images of DAPI-stained samples showed that no nuclear components were visible after decellularization. SEM imaging indicated that all samples contained well-aligned collagen fibers that became tighter after the attachment of GA-Ag NPs. Compared with NT, DT featured a significantly lower DNA content (Fig. 2b; 202.3 ± 25.29 ng/mg vs. 9.005 ± 3.695 ng/mg, *p* < 0.0001) but featured a similar collagen content (Fig. 2c; 417.2 ± 24.04 μg/mg vs. 363.6 ± 66.36 μg/mg, *p* > 0.05). EDS analysis (Figs. 2d and e) indicated the presence of Ag in DT-Ag but not in DT, further showing that Ag in DT-Ag was abundant and evenly distributed (Fig. 2f). TEM imaging showed the presence of nanoscale substances on the surface of collagen fibers (Fig. 2g). Taken together, the above results indicated that GA-Ag NPs were successfully deposited on the collagen fibers of the tendon and were chemically grafted on the fiber surface through EDC/NHS coupling. Figure 3a presents the results of zeta potential measurements. GA-Ag NPs had a notable negative charge (− 9.84 ± 1.88 mV), while both DT and DT-Ag featured positive charges (9.263 ± 2.14 mV and 2.01 ± 2.08 mV, respectively). The smaller positive charge of DT-Ag compared to that of DT was ascribed to the loaded GA-Ag NPs. Bacterial cell membranes are known to be negatively charged because of the presence of carboxyl and phosphate groups [39]. The electrostatic repulsion between GA-Ag NPs and microbial cell membranes can inhibit bacterial adhesion [33] and thus increase the antibacterial activity. The FTIR spectra of DT and DT-Ag (Fig. 3b) featured typical amide C = O and NH bands around 1640 and 2910 cm^−1^, respectively [38]. These peaks lost intensity after the introduction of the carboxyl group–bearing GA, which confirmed amide bond formation and the influx of numerous carboxyl groups. The X-ray photoelectron spectrum of DT-Ag, unlike that of DT, featured Ag 3d signals (Fig. 3c). The Ag 3d spectrum of DT-Ag (Fig. 3d) was deconvoluted into two peaks at 368.18 and 374.08 eV, which corresponded to Ag 3d_5/2_ and Ag 3d_3/2_ transitions, respectively, indicating that silver mainly existed in elemental form. The distance between these peaks, i.e., the spin–orbit coupling (~ 6.0 eV), implied the formation of metallic silver on the collagen fiber surface [40, 41].

The modification of Ag NPs with GA was thought to change their hydrophilicity. Moreover, hydrophilicity could have been further increased by the attachment of GA-Ag NPs to collagen fibers on micro- and nanoscales and the resulting changes in the fiber surface roughness. Figure 3e shows that the water contact angle of DT-Ag (62.69 ± 0.56°) was lower than that of DT (71.61 ± 0.41°), indicating that the former material was more hydrophilic, probably because of the anionic and hydrophilic carboxyl group of GA. The improved hydrophilicity of GA-Ag NPs may not only result in better wetting, but also provide the biological antifouling ability and reduce fibrin deposition [20–23]. The fibroblast adhesion experiment (Fig. 3f) proved that numerous cells adhered to the porous plate used as a control, whereas the living cells on the surfaces of DT and DT-Ag were much less numerous and less densely distributed.

Mechanical properties were determined by tensile testing, which was performed on an electronic universal testing machine. Both ends of the tendon were held with sandpaper, and the sample was broken in the middle. Figure 3g presents the stress–strain curves obtained during mechanical property testing, while Figs. 3h&i show ultimate stresses and failure strains, revealing that they did not significantly vary among the samples.

The carboxylic acid groups of GA-Ag NPs allow for chemical attachment to collagen fibers via EDC/NHS coupling. The UV absorbance and quantity changes of the GA-Ag NP solution were tested before and after attachment to determine attachment efficiency. Figure 4a presents the concentration–absorbance plot constructed using the results of ICP-OES and enzyme marker analyses, revealing that this plot was well linear within the GA-Ag NP absorbance range of 0.038–2.4. Figure 4b reveals that the absorbance of the GA-Ag NP solution and hence, the total amount of GA-Ag NPs, did not significantly change after soaking. The materials were randomly divided into GA-Ag NP–grafted and nongrafted groups for subsequent study.

According to Fig. 4c, the initial amount of GA-Ag NPs was close to 112 μg at a DT loading of 50 mg, [EDC] = [NHS] = 0 mM, and a GA-Ag NP concentration of 700 μg/mL. After 6-h oscillation, the amount of GA-Ag NPs remaining in the solution was 58.34 ± 3.28 μg. According to the formula in Sect. 2.5.2, *M*_load_ was 53.66 ± 3.28 μg, and *χ*_load_ was 47.91 ± 2.93%. At [EDC] = 2[NHS] = 40 mM, only 3.43 ± 0.94 μg of GA-Ag NPs was collected in the supernatant after 6-h grafting, and *M*_load_ and *χ*_load_ were determined as 108.57 ± 0.94 μg and 96.93 ± 0.84%, respectively. After continuous ultrasonication, the content of GA-Ag NP residues on the tendon was calculated and recorded. After five washing cycles, the residual contents of GA-Ag NPs in both groups were stable and nearly unchanged. At this point, the difference between the two groups (line a, Fig. 4c) could be considered as the relative content of GA-Ag NPs attached to the tendon, and *χ*_attachment_ was calculated as 65.47 ± 0.57% (line a/b, Fig. 4c). The supernatant collected after the first ultrasonic circulation of DT-Ag was collected and shown to contain scattered GA-Ag NPs by TEM imaging (Fig. 4d).

### Release performance of GA-Ag NPs

DT-Ag appeared to be dense GA-Ag NP–collagen fiber complexes that release GA-Ag NPs and provide long-lasting antibacterial activity by killing bacteria adhered to the tendon surface. The release behavior of GA-Ag NPs in DT-Ag was studied using an enzyme labeling method. Figure 4e shows the release of GA-Ag NPs in the nongrafted ([EDC] = [NHS] = 0 mM) and grafted ([EDC] = [2NHS] = 40 mM) groups. The cumulative content of GA-Ag NPs released in the nongrafted group was 50.3 ± 6.29% at 7 days, while that in the grafted group was only 22.29 ± 2.07% (*p* < 0.0001).

Figure 4f shows that after seven ultrasonication-assisted washing cycles, the cumulative contents of GA-Ag NPs released in nongrafted and grafted groups were 49.78 ± 6.29% and 9.74 ± 0.92%, respectively (*p* < 0.0001). These results indicate that GA-Ag NP release exhibited an extremely persistent behavior, possibly because of the action of the coupling agent. Thus, the novel GA-Ag NP–grafted tendon may provide long-lasting antibacterial activity.

### Antibacterial activity

As shown in Figs. 4g and h, DT did not form an inhibitory zone, whereas DT-Ag inhibited the proliferation of both *S. aureus* (*D* = 6.97 ± 2.13 mm) and *E. coli* (*D* = 4.82 ± 1.22 mm). The spread-plate method was used to further evaluate the bactericidal behavior of DT-Ag against plankton bacteria. After 18-h coculturing with the bacterial solution, the number of bacterial colonies in the DT-Ag group was much less than that in the DT group (Fig. 4i, Fig. S2b). These results indicated that DT-Ag had good antibacterial activity against both bacterial strains.

The morphology of the bacteria on DT and DT-Ag was observed by SEM. As presented in Fig. 4j, numerous *S. aureus* (yellow) and *E. coli* (green) bacteria were present in the DT group, whereas only a few bacteria were detected in the DT-Ag group, and an incomplete cell shell was observed on the DT-Ag surface (blue arrow). PI staining experiments (Fig. S3) revealed the presence of numerous dead bacteria on the surface of DT-Ag, whereas almost no dead bacteria were observed on the surface of DT. This finding indicated that the ordinary tendon had no bactericidal effect, which was consistent with the results of bacteriostatic zone measurements. On the one hand, DT-Ag was decorated by numerous negatively charged GA-Ag NPs, which could repel dead bacteria. On the other hand, the loaded GA-Ag NPs exhibited high bactericidal activity, which was consistent with the results of SEM imaging (Fig. 4j).

## Biocompatibility

Figures 5a and b present the cytotoxicities of DT and DT-Ag determined using the CCK-8 assay. No significant differences were observed between the control group (NIH 3T3 cultured with DMEM) and other groups (NIH 3T3 cultured with 25, 50, 75, and 100% DMEM extracted from DT-Ag) at all time points.

Normal saline (NS) and double distilled water (H_2_O) were used as negative and positive controls, respectively, in the hemolysis test (Fig. 5c). In NS, DT-Ag, and DT groups, the red blood cells sank, and the upper fluid was colorless and transparent, while in the H_2_O group, the red blood cells ruptured, i.e., hemolysis occurred (Fig. 5c). The quantitative analysis of solution OD revealed the same trend (Fig. 5d).

### In vitro antioxidant and anti-inflammatory activities

Given that GA exhibits anti-inflammatory and antioxidant activities [42], we tested DT-Ag for the same. Figure 6a presents the DPPH radical scavenging activities of ascorbic acid, GA, DT, and DT-Ag. Ascorbic acid maintained high antioxidant activity in the range of 10–150 μg/mL, whereas the activity of GA increased with increasing concentration and was close to 80% at 30 μg/mL. Notably, the activity of DT-Ag showed the same trend as that of GA, exceeding 60% at an initial concentration of 50 μg/mL, while DT showed low antioxidant activity. Thus, GA-Ag NPs endowed DT-Ag with high antioxidant activity.

Figure 6b presents the results of the anti-inflammatory activity evaluation, demonstrating that DT-Ag had a certain anti-inflammatory activity that exceeded that of GA at high concentrations. Compared with DT, DT-Ag showed much higher anti-inflammatory activity.

### Macrophage polarization performance

Macrophages of the M1 subtype are thought to release IL-1β and specific surface markers (such as iNOS and CCR7) during inflammatory responses [43]. In contrast, M2 macrophages produce anti-inflammatory cytokines and proteins (e.g., IL-4 and CD206) contributing to the establishment of an anti-inflammatory microenvironment [44]. Therefore, macrophages, as major inflammation responders, are becoming important players in pathogen eradication and tissue repair [45].

M1 (inducible nitric oxide synthase, iNOS)- and M2 (CD206)-labeled macrophages were identified by immunofluorescence analysis [46] to study the effect of DT-Ag on the polarization of RAW 264.7 macrophages after a predetermined coculturing time. As demonstrated in Fig. 6c, the LPS-induced M1 positive group showed a higher percentage of iNOS-positive cells and a lower percentage of CD206-positive cells, while the IL-4-induced M2 positive group showed a higher percentage of CD206-positive cells. CD206-positive cells in the DT-Ag group were significantly more abundant than those in the DT group.

The mRNA expression levels of M1 and M2 macrophage-related markers including iNOS, IL-1β, CD206, IL-10, and Arg-1 were further verified by qRT-PCR (Fig. 6d). The secretion of the pro-inflammatory factor (iNOS) of M1 markers did not increase in all groups and decreased with the increasing amount of GA-Ag NPs. The release of anti-inflammatory factors was promoted in all groups except the control group. The expression of M2 markers (CD206, IL-10, Arg-1) increased upon the addition of GA-Ag NPs at appropriate concentrations. These results showed that at a suitable concentration, GA-Ag NPs can promote macrophage conversion to the M2 type. At an overly high concentration, the cytotoxic GA-Ag NPs may reduce the number of live macrophages and their M2 polarization, which agrees with the results of Yang et al. [19].

### In vivo experiments

**Fig. 7:**
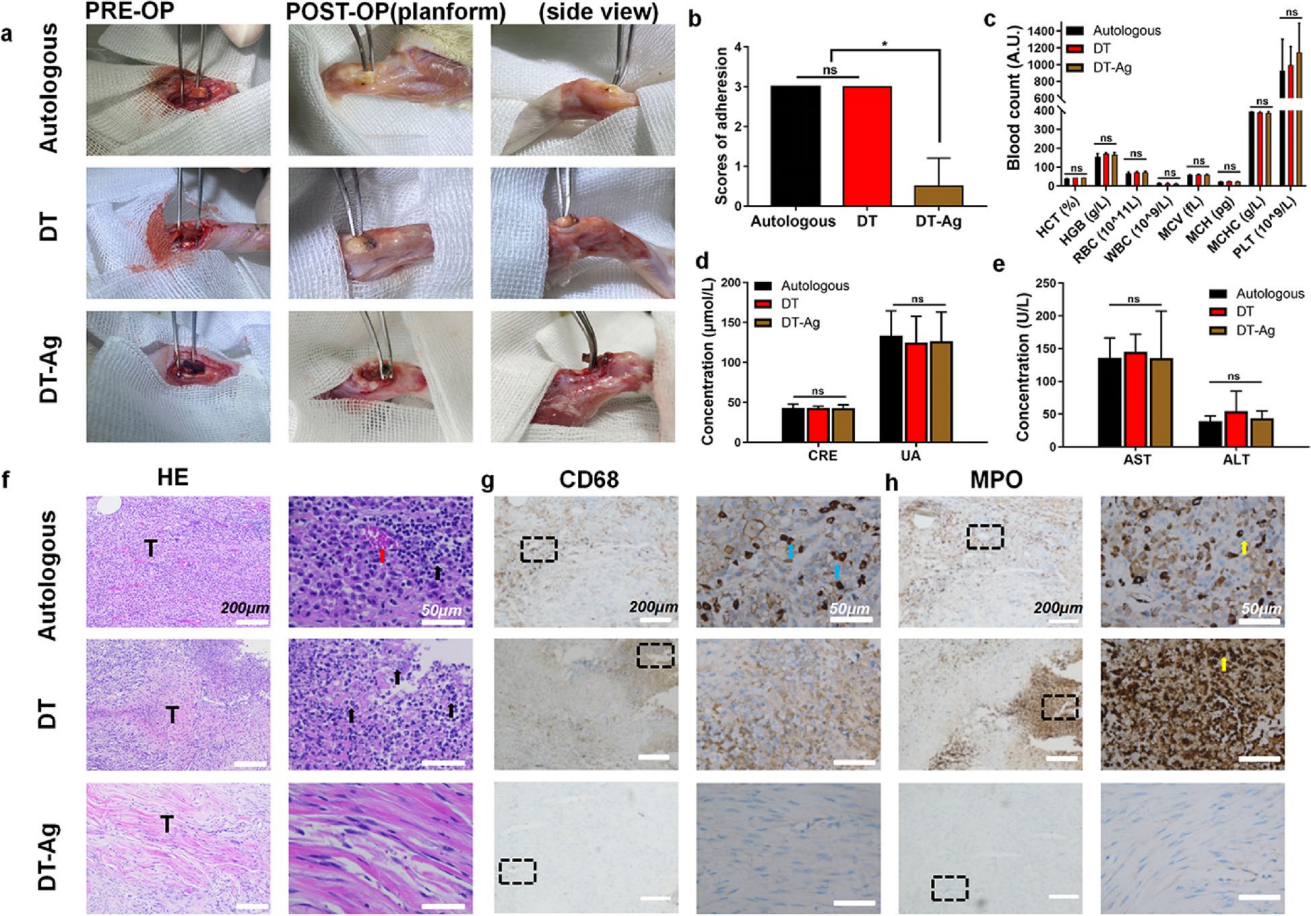
Antibacterial, antiadhesion, and anti-inflammatory activities of DT and DT-Ag in vivo. **a** Tendon adhesion at three weeks after AT, DT, and DT-Ag implantation. **b** Results of adhesion grade analysis. **c** Blood routine, (**d**) renal function, and (**e**) liver function determined one week after implantation for toxicity analysis. Results of staining performed three weeks after implantation: (**f**) H&E staining images of inflammatory cells (black arrow for inflammatory cells, red arrow for red blood cells), (**g**) CD68 staining images of macrophages (blue arrow), and (**h**) MPO staining images of neutrophils (yellow arrow). *: *p* < 0.05

A model of a defective rat Achilles tendon was used to study the antiadhesion properties of DT-Ag in vivo. According to the graft used, the rats were divided into autologous tendon (AT), DT, and DT-Ag groups. Figure 7a shows the adhesion observed for each group after surgery (separation was performed using bending tweezers). The AT group was characterized by severe adhesion and the inability to perform blunt dissection by the bending tweezers. In the DT group, we observed a possibly severely infected swollen tendon that could not be separated from the surrounding tissues. In the DT-Ag group, blunt separation was possible with the slight help of bending tweezers. The adhesion scores presented in Fig. 7b show that inflammatory adhesion was observed in AT and DT groups, whereas only slight adhesion (i.e., antiadhesion activity) was observed in the DT-Ag group.

No significant differences in the whole blood cell count and liver/kidney function were observed among all groups one week after the operation (Figs. 7c–e), which suggested that new tendons do not cause significant inflammatory reactions or metabolic abnormalities.

Three weeks after the operation, the rats were euthanized, and the tendons were removed for histological analysis. The imaging of H&E-stained samples (Fig. 7f) showed that numerous inflammatory cells and some red blood cells were observed in the AT group. This finding indicated that the AT was still in the process of healing and inflamed, which facilitated adhesion to adjacent tissues and precluded passive separation. In the DT group, unlike in the other two groups, numerous lymphocytes and neutrophils as well as necrotic tissues were observed. Therefore, the tendon swelling observed in this group was indicative of serious inflammation, which led to tendon necrosis and aggravated adhesion. However, no inflammatory cells were found in the DT-Ag group. The further immunohistochemical staining of macrophages (Fig. 7g) and neutrophils (Fig. 7h) showed that both cell types were present in the AT group. In the DT group, significant neutrophil infiltration but no macrophages were observed, which indicated that the tendon was still in the stage of acute infection. However, no obvious inflammatory cell infiltration was observed in the DA-Ag group, which may have been controlled in the early stage of infection, indicating that the new tendon played a significant role in the two important processes (anti-inflammation and antiadhesion) of postoperative healing.

## Discussion

In order to obtain the antibacterial and anti-adhesion performance of implanted tendon, some scholars have carried out the relevant research. For example, *chen *et al*.* assembled silver nanoparticles (Ag NPs), polylactic acid and hyaluronic acid into nanofiber membranes. Ag NPs and hyaluronic acid play the role of antibacterial and anti-adhesion, respectively [47]. Zhang et al. prepared an oxidative stress-responsive electrospun polyester membrane loaded with curcumin and celecoxib, which could respond to oxidative stress. The curcumin is used for bacteriostasis, while the celecoxib is used for anti-inflammatory and anti-adhesion [7]. *Shalumona *et al*.* prepared core–shell nanofibrous membranes with core/shell structure by Ag NPs, ibuprofen and hyaluronic acid. Ag NPs located in the shell for sterilization, while ibuprofen and hyaluronic acid located in the core for anti-inflammatory and anti-adhesion, achieving a double effect [12]. However, these above studies were limited to the preparation of antibacterial and anti-adhesion membranes to cover the surface of the tendon, which possess the following defects for clinical use. Firstly, the membrane suture was required to be fixed on the tendon during application, and the suture operation would cause changes in the structural characteristics of the barrier, leading to adverse inflammation. Secondly, the sutures and membranes may hinder tendon sliding. Thirdly, since the size of the tendon defect varies from patient to patient, the membrane needs to be trimmed during surgery to accommodate the injury site. Therefore, it is particularly important to prepare a kind of tendon graft with dual functions of antibacterial and anti-adhesion.

GA-Ag NPs with a size of 8.38 ± 0.77 nm were synthesized via using gallic acid as a protective agent. The bactericidal capacity of Ag NPs is known to be negatively correlated with their size [14]. Therefore, maintaining the size and stability of GA-Ag NPs is crucial for ensuring effective bactericidal activity. The UV absorption peak of GA-Ag NPs remained unchanged for 46 days, indicating their excellent stability. Our previous research has demonstrated that infection causes a slight alteration in the pH of the internal environment, as measured by blood gas analysis, with a value of 6.1 ± 0.6 [38]. The UV absorption intensity of GA-Ag NPs remained stable within the pH range of 4 to 11, demonstrating their pH stability in this range. However, when pH value was set at 3, the UV absorption intensity changed, and further TEM observation revealed agglomeration of GA-Ag NPs, leading to variations in their sizes. It is important to note that the particle size of GA-Ag NPs measured by DLS is larger. For instance, at the pH = 6, the particle size is approximately 22.01 ± 0.87 nm, which is still less than that of at pH = 3 (29.47 ± 0.43 nm). This increase in particle size may be attributed to the abundant negatively charged carboxyl groups present in gallic acid, the protective agent of GA-Ag NPs [33].

We then observed good antibacterial activity of GA-Ag NPs against two representative bacteria, both Gram-positive (Staphylococcus aureus) and Gram-negative (Escherichia coli). To establish the relationship between absorbance and concentration, we combined data from ICP-OES and UV spectrum analysis to draw an "absorbance-concentration curve." In order to ensure accuracy, we restricted the range of absorbance values to minimize errors. At lower concentrations, a more pronounced effect against E. coli was observed, while as the concentration increased, the difference became less noticeable. The current understanding of the antibacterial mechanism suggests a synergistic effect between Ag NPs and Ag ions [39]. It is worth noting that the variation in antibacterial effectiveness may be attributed to the thicker cell walls of gram-positive bacteria.

Subsequently, GA-Ag NPs were loaded by using two methods: electrostatic absorption and chemical crosslinking. These methods were confirmed through Zeta potential and infrared spectrum analysis. GA serves as a protective agent, and its hydroxyl groups on the surface can chelate with silver ions, resulting in the formation of negatively charged GA-Ag NPs through chemical reduction. The change in Zeta potential and the absorption peak at approximately 1640 cm^−1^ in the infrared spectrum provided evidence for the two different synthesis methods of GA-Ag NPs. Acellular organism tissues typically contain a significant amount of positive charge [38], which can interact with the negatively charged GA-Ag NPs. The loaded GA-Ag NPs exhibited a slow release under ultrasonic shock and agitation conditions, with approximately 50.3 ± 6.29% released within a week. The amino groups exposed by DT can be cross-linked with the carboxyl groups exposed on GA-Ag NPs, forming amide bonds [35]. The introduction of GA carboxyl groups led to a decrease in the peaks at 2910 cm^−1^ and 1640 cm^−1^, confirming the formation of amide bonds and the incorporation of a large number of carboxyl groups. After the crosslinking process, the loaded GA-Ag NPs increased significantly, and their release under agitation or ultrasonic shock was longer and slower. After one week, the total GA-Ag NPs release from the crosslinking group was only 22.29 ± 2.07% of the initial dose, indicating a substantial amount of GA-Ag NPs still present within DT-Ag, thereby maintaining long-term antibacterial effects.

After tendon injury, the acute inflammatory response initiates [5, 7], characterized by increased secretion of pro-inflammatory factors and infiltration of circulating inflammatory cells. This inflammatory process leads to enhanced exudation at the injury site, exacerbating fibrin leakage and increasing the risk of tendon adhesion. Fibroblasts [48, 49], which are resident cellular components of the tendon sheath and synovium, become activated during the inflammatory response. They secrete a significant amount of collagen in the injured area, promoting tendon adhesion to surrounding tissues. Additionally, they form a matrix with chemotactic properties, stimulate fibroblast proliferation and migration, and further contribute to tendon adhesion. To address the adhesion of fibroblasts, different treatments were compared. We observed a significant decrease in cell adhesion after DT-Ag group. This intervention could potentially alleviate tendon adhesion caused by excessive fibroblasts. Kim H et al. [45] discovered that macrophages, as key participants in the inflammatory response, play crucial roles in pathogen elimination and tissue repair. Specially modified GA-Ag NPs [19] can actively target M1 macrophages to induce their reduction and polarization into M2 macrophages, thus regulating inflammation. By the way, the GA-Ag NPs investigated in this research can be substituted with other kind of materials possessing similar traits, but the author has not identified such alternatives yet. Further in-depth research is required to explore and discover suitable replacements.

We investigated the effects of acellular tendons loaded with gallic acid-modified Ag NPs on macrophage behavior (RAW264.7). After one day of co-culture, we observed that a certain concentration of GA-Ag NPs reduced the expression of M1 markers in macrophages, while the group containing GA-Ag NPs promoted the expression of M2 markers. Immunofluorescence analysis revealed a more pronounced M2 polarization trend in DT-Ag compared to the positive group (IL-4) and the DT group. Furthermore, we confirmed the anti-inflammatory capability through erythrocyte membrane stability, which exhibited a significant improvement after loading GA-Ag NPs in acellular tendons. The DPPH method yielded similar results, indicating enhanced anti-inflammatory properties. Ag NPs are usually associated with cytotoxicity through oxidative stress-induced apoptosis and cellular component damage [50]. Antioxidants can minimize the cytotoxicity of Ag NPs [14], and DT-Ag has good antioxidant properties, which may be related to the strong negative charge of gallic acid in GA-Ag NPs, which can combine with DPPH to remove oxygen free radicals. DT-Ag was chemically cross-linked with GA-Ag NPs, which showed slow and persistent release in the release experiment. We extracted the soaking solution and co-cultured fibroblasts, and detected at a specific time, almost no cytotoxicity was found.

Acellular tendons have the ability to preserve the natural spatial structure of tendons and retain certain factors within the extracellular matrix that can induce the growth and differentiation of muscle satellite cells [28]. This characteristic greatly promotes the regeneration of homologous tissues. In a rat model of tendon defect, we observed that the tendon adhesion score in the DT-Ag treatment group, was significantly lower than that of the autologous tendon (AT) group and the DT group. Histological examinations using H&E staining and immunohistochemistry revealed the absence of inflammatory cells in the DT-Ag treatment group, with a well-organized tissue structure. Furthermore, blood reports of the three groups showed almost no signs of cytotoxicity, which aligns with the findings from in vitro experiments.

## Conclusion

GA-Ag NPs with an average size of 8.38 ± 0.77 nm, excellent colloidal stability, and high antibacterial activity were attached to collagen fibers via EDC/NHS coupling and formed micro- and nanoscale bilayer structures, thus endowing the corresponding tendon (DT-Ag) with good bactericidal properties and increased hydrophilicity. The bilayer structures efficiently suppressed the proliferation of both Gram-positive and Gram-negative bacteria, which was attributed to the slow release of antimicrobial silver NPs and the electrostatic repulsion between bacterial cell walls and the GA-Ag NP layer. Furthermore, the antioxidant, anti-inflammatory, and antiadhesion activities of DT-Ag were confirmed by in vitro and in vivo tests. The successful preparation of this new type of tendon with antibacterial and antiadhesion activities may provide new insights and treatment options for tendon reconstruction surgery.

### Supplementary Information

**Additional file 1.**

## Data Availability

The datasets used and/or analyzed during the current study are available from the corresponding author on reasonable request.

## Abbreviations

GA: Gallic acid
Ag NPs: Silver nanoparticles
AT: Autologous tendon
DT: Decellularized Tendon
DT-Ag: Decellularized Tendon crosslinking AgNPs
ECM: Extracellular matrix
EDC: 1-(3-Dimethylaminopropyl)-3-ethylcarbodiimide hydro
NHS: N-Hydroxy succinimide
PBS: Phosphate Buffered Saline
S.aureus: Staphylococcus aureus
E.coli: Escherichia coli
CCK8: Cell Counting Kit-8
DPPH: 2,2-Diphenyl-1-picrylhydrazyl
H&E: Hematoxylin and eosin staining
DAPI: 4′6-Diamino-2-phenylindole staining
TEM: Transmission electron microscopy
SEM: Scanning electron microscopy
EDS: Energy-dispersive spectroscopy
XPS: X-ray photoelectron spectroscopy
OD: Optical density
