# Supplementary material for: Silver nanoparticles–decorated extracellular matrix graft: fabrication and tendon reconstruction performance
Source: Biomater Res. 2023 Sep 14;27:85. doi: 10.1186/s40824-023-00428-0 (PMC10503197; doi:10.1186/s40824-023-00428-0)
Supplement: Supplementary file 1 — Additional file 1. [file 40824_2023_428_MOESM1_ESM.docx]

**Silver nanoparticles–decorated extracellular matrix graft: fabrication and** **tendon reconstruction performance**

Sunfang Chen^1,2#^, Dan Cai^3#^, Qi Dong^4#^, Gaoxiang Ma^2^, Chennan Xu^2^, Xiaogang Bao^5^, Wei Yuan^6*^, Bing Wu^2*^, Bin Fang^1,2*^

1 Department of Orthopedics, the First Affiliated Hospital of Zhejiang Chinese Medical University (Zhejiang Provincial Hospital of Chinese Medicine), Hangzhou, 310000, China.

2 Department of Orthopedics, the Central Hospital Affiliated to Shaoxing University, Shaoxing, 312030, China.

3 Department of Orthopedics, the First People’s Hospital of Huzhou, First Affiliated Hospital of Huzhou University, Huzhou,313000, China.

4 Department of Orthopedics, Honghui Hospital, Xi'an Jiao Tong University, Xi'an City, 710054, China.

5 Department of Orthopedics, The Spine Surgical Center, Second Affiliated Hospital of Naval Medical University, Shanghai, 200003, China.

6 Department of Orthopedics, Shanghai Municipal Hospital of Traditional Chinese Medicine, Shanghai University of Traditional Chinese Medicine, Shanghai, 200071, China.

^#^These authors contribute equally.

*Corresponding author.

E-mail: Wei Yuan (wei95960@126.com), Bing Wu (wubing@usx.edu.cn), Bin Fang (20233006@zcmu.edu.cn)

**1. Materials**

Sodium borohydride (NaBH4, ＞96%) was purchased from Aladdin Co., Ltd. (Shanghai, China). Silver nitrate, GA, propidium iodide (PI), calcein, *N*-hydroxysuccinimide (NHS), 1-ethyl-3-(3-dimethylaminopropyl) carbodiimide (EDC) and NIH3T3 was purchased from Solarbio Biotech Co., Ltd. (Beijing, China). Triton X-100, and sodium dodecyl sulfonate were purchased from Sinopod Chemical Reagent Co., Ltd. (China). The hydroxyproline test kit was procured from Beijing Sun Biotechnology Co., Ltd. (China). Agar was purchased from Sangon Biotech Co., Ltd. (Shanghai, China), tryptone was obtained from Oxoid (Shanghai, China), and soybean peptone was provided by Sinophosphoric Chemicals Co., Ltd. (China). The DNA kit was procured from CW Biotech (Beijing, China) and the Cell Counting Kit-8 (CCK-8) was procured from APExBIO Technology LLC (USA). *Escherichia coli* (ATCC25922) and *Staphylococcus aureus* (ATCC6538) was purchased from American type culture collection (ATCC, America).

**2. Characterization of** **GA-Ag NP**

The absorption spectra of GA-Ag NP solutions (100 μL) with different concentrations were measured at 200-600 nm to construct an optical density–concentration curve. GA-Ag NP solutions (10 mL) of different concentrations were analyzed by inductively coupled plasma-atomic emission spectrometry (ICP-OES; Thermo Fisher iCAP PRO (OES), America). GA-Ag NP solutions evaporate, dissociate, ionize and get excited in the excitation light source to produce light radiation. The resultant composite light is dispersed into a spectrum by spectroscopic dispersion. The wavelength and intensity of spectral lines are detected and analyzed. The pH-dependent size distribution of GA-Ag NPs was analyzed by transmission electron microscopy (TEM; JEM-1011, JEOL, Japan) and dynamic light scattering (Malvern Zetasizer Nano ZS90, Britain).

**3. Characterization of DT decorated with GA-Ag NPs (DT-Ag)**

**Histological analysis and** **mechanical property evaluation of DT-Ag**

DT-Ag was pruned to a suitable size for H&E and DAPI staining. Another part of DT-Ag was sputter-coated with Au-Pd and observed by SEM. The Instron tension system was used to test the tension of DT-Ag according to the method described above.

**Morphology and elemental composition analysis**

The morphologies of DT and DT-Ag and the distribution of Ag in samples were observed by SEM coupled with energydispersive spectrometry analysis (X-Act, OXFORD, British). The chemical state of Ag in DT-Ag was further analyzed by X-ray photoelectron spectroscopy (Thermo Scientific K-Alpha, USA).

**Zeta potential measurements**

DT and DT-AG were freeze-dried and repeatedly ground into powder using a pulverizer (JXFSTRP-24, Shanghai China). The sample was nitrated in concentrated nitric acid. Zeta potentials were then measured using a Malvern Zetasizer Nano Z instrument (UK).

**Functional group analysis**

The functional groups of freeze-dried and ground GA-Ag NPs, DT, and DT-Ag were measured between 3800 and 600 cm^–1^ and analyzed by Fourier transform infrared (FTIR) spectroscopy (Thermo Scientific Nicolet iS2).

**Determination of antiadhesion activity in vitro**

For cell adhesion measurements, DT and DT-Ag were cut into 100μm thin slices, sterilized under ultraviolet (UV) light for 4 h, placed in 24-well plates, and inoculated with NIH-3T3 cells at a density of 2 × 10^4^ cells/well for *in vitro* cell culturing. The cell medium DMEM (Dulbecco's modified eagle medium) (1 mL) containing 10 vol% FBS (Fetal Bovine Serum) and 1 vol% antibiotic/antifungal agent were added to each well, and the cells were kept in a 5 vol% CO_2_-filled incubator humidified at 37 °C. Cells inoculated in 24-well plates in the absence of DT and DT-Ag were used as controls. On day 3, the samples were stained with calcein AM (2 μM) and incubated for 30 min at 37°C under a fluorescence microscope.

**The hydrophilicities test**

The surface hydrophilicities of DT and DT-Ag were quantified in terms of the respective water contact angles (JY-82B Kruss DSA), which were determined as the averages of measurements performed at three different points on each surface.

**4. Cytotoxicity test**

Cytotoxicity was determined using the CCK-8 method. DT and DT-Ag were sterilized by irradiation at a dose of 25 kGy and then soaked in DMEM at 37 °C (sample volume/medium volume = 1:5) for 48 h. The obtained supernatant was collected for further use. Mouse fibroblasts (NIH-3T3) were inoculated into 96-well plates at a density of 1000 cells per well. After the cells had been cultured at 37 °C for 24 h, the supernatant was removed, and 100 µL gradient concentration of extract (0, 25, 50, 75, or 100% (v/v), *n* = 3) was added to each well for 1, 3, and 5 days, respectively. Then, the CCK-8 reagent (10 µL) was added to each well, and the wells were incubated at 37 °C for 1 h to quantify cell metabolism. The absorbance at 450 nm was measured as above.

**5. Macrophage polarization analyse**

**Immunofluorescence method**

M1 (iNOS) and M2 marker (CD206)-expressing macrophages were analyzed by an immunofluorescence method. RAW 264.7 cells were cocultured with scaffold (1 × 10^5^ cells/well) for 1 day. The macrophages were then scraped off and seeded on a new plate for reattachment, which was followed by fixation with paraformaldehyde/PBS (4 vol%), permeabilization with Triton X-100, and closure with 5% normal goat serum for 1 h. Next, the specimens were incubated with primary antibodies (ab210823 and ab64693) overnight at 4 °C. Secondary antibodies (ab6785 and ab6939) were incubated with the sample for 1 h. After restaining with DAPI for 10 min, the cells were detected by confocal laser scanning microscopy (CLSM, TCS SP2; Leica, Wetzlar, Germany). Lipopolysaccharide (LPS) induction (200 ng/mL) was used as the M1 positive control, while IL-4 induction (20 ng/mL) was used as the M2 positive control.

**Reverse transcription-quantitative polymerase chain reaction (RT-qPCR)**

RAW 264.7 cells were inoculated on the sample at a density of 1 × 10^5^ cells/well. After 1-day incubation, RNA was collected using a Yew kit, and reverse transcription was performed using a reverse transcription kit (Yew) according to manufacturer's instructions. The expression levels of the housekeeping gene GADPH were used to normalize those of the genes of interest. The primers used for the real-time qPCR are listed in Table 1.

**Table S1.** Primer sequences used for real-time qPCR.

| **Gene** | **Forward primer (5′→3′)** | **Reverse primer (5′→3′)** |
| --- | --- | --- |
| IL-1β | ATGAAGGGCTGCTTCCAAAC | TCTCCACAGCCACAATGAGT |
| iNOS | CCTGGTGCAAGGGATCTTGG | GAGGGCTTGCCTGAGTGAGC |
| Arg-1 | TGGCTTGCGAGACGTAGAC | GCTCAGGTGAATCGGCCTTT |
| IL-10 | CTGGACAACATACTGCTAACCG | GGGCATCACTTCTACCAGGTAA |
| CD206 | GGTTCCGGTTTGTGGAGCAG | TCCGTTTGCATTGCCCAGTA |
| GAPDH | GGGTCCCAGCTTAGGTTCAT | CCAATACGGCCAAATCCGTT |

**6. Statistical analysis**

Data were expressed as means ± standard deviations. The univariate analysis of variance and the paired T-test implemented into GraphPad Prism 2021 software (GraphPad Software Inc., USA) were used for data analysis. The sample size was *n* = 3 in all cases, and significance was defined as *p* < 0.05.

**7. Ethical approval**

The animal experiments were conducted at the Laboratory Animal Center of Zhejiang Academy of Medical Sciences in accordance with the National Institute of Health Guidelines and the Zhejiang Academy of Medical Sciences Guidelines for the Care and Use of Laboratory Animals.


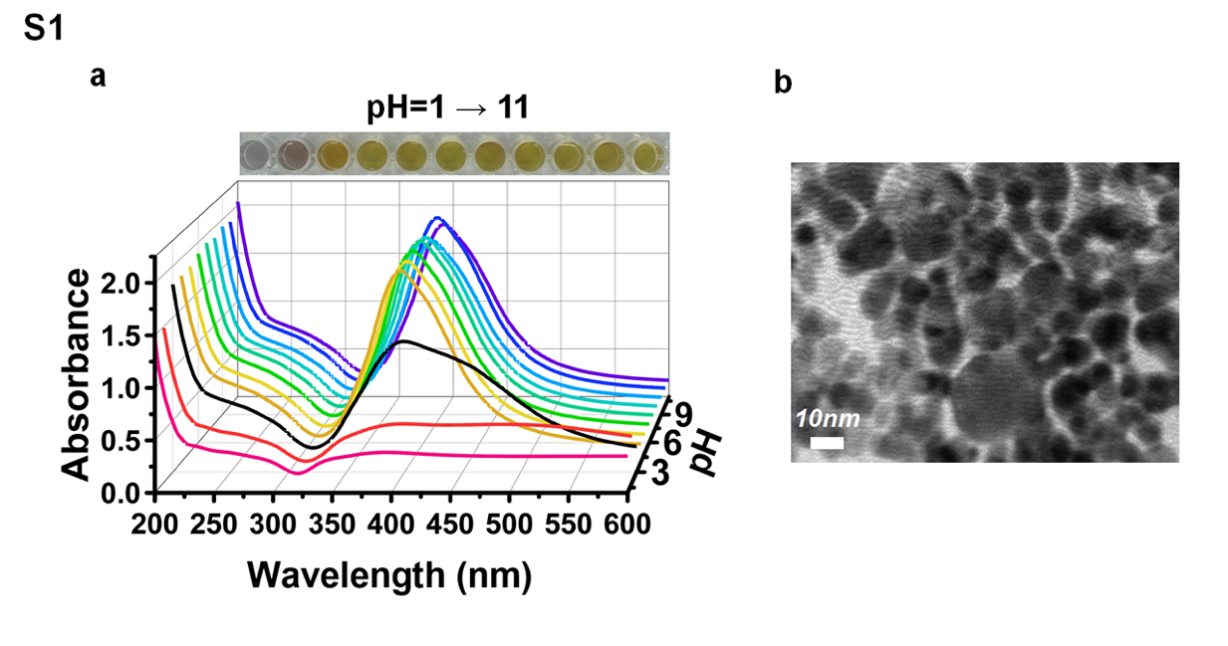


**Fig. S1** (a) Absorption spectra of GA-Ag NPs at pH=1-11 and (b) TEM image of GA-Ag NPs at pH=3

**
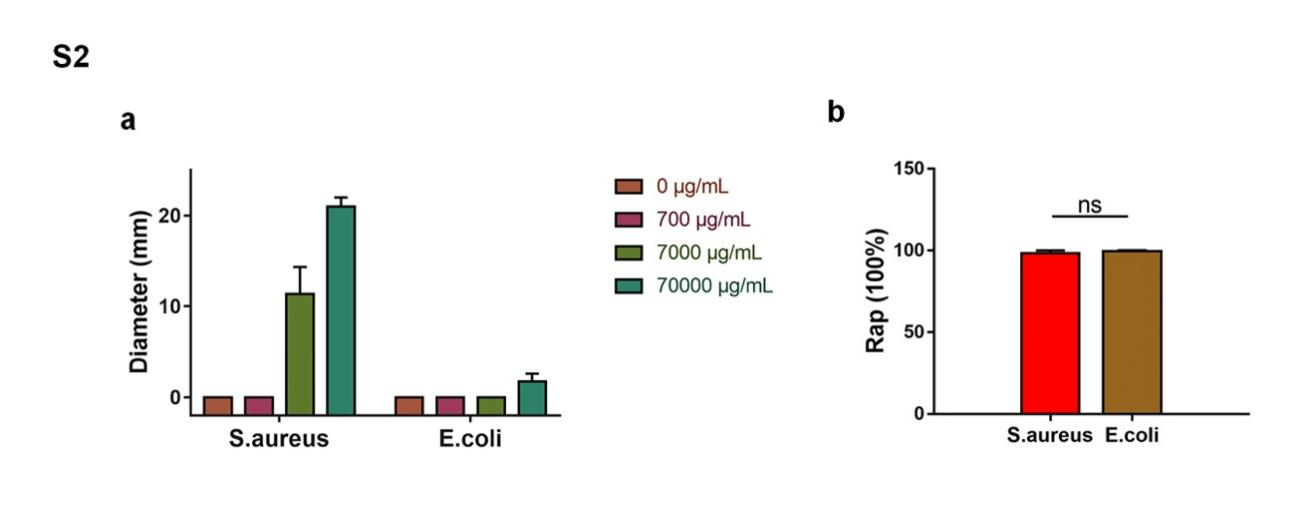
**

**Fig. S2** (A) Inhibitory zone size analysis of GA-AgNPs in Staphylococcus aureus and Escherichia coli, and (B) Inhibitory zone size analysis of DT-Ag in Staphylococcus aureus and Escherichia coli.


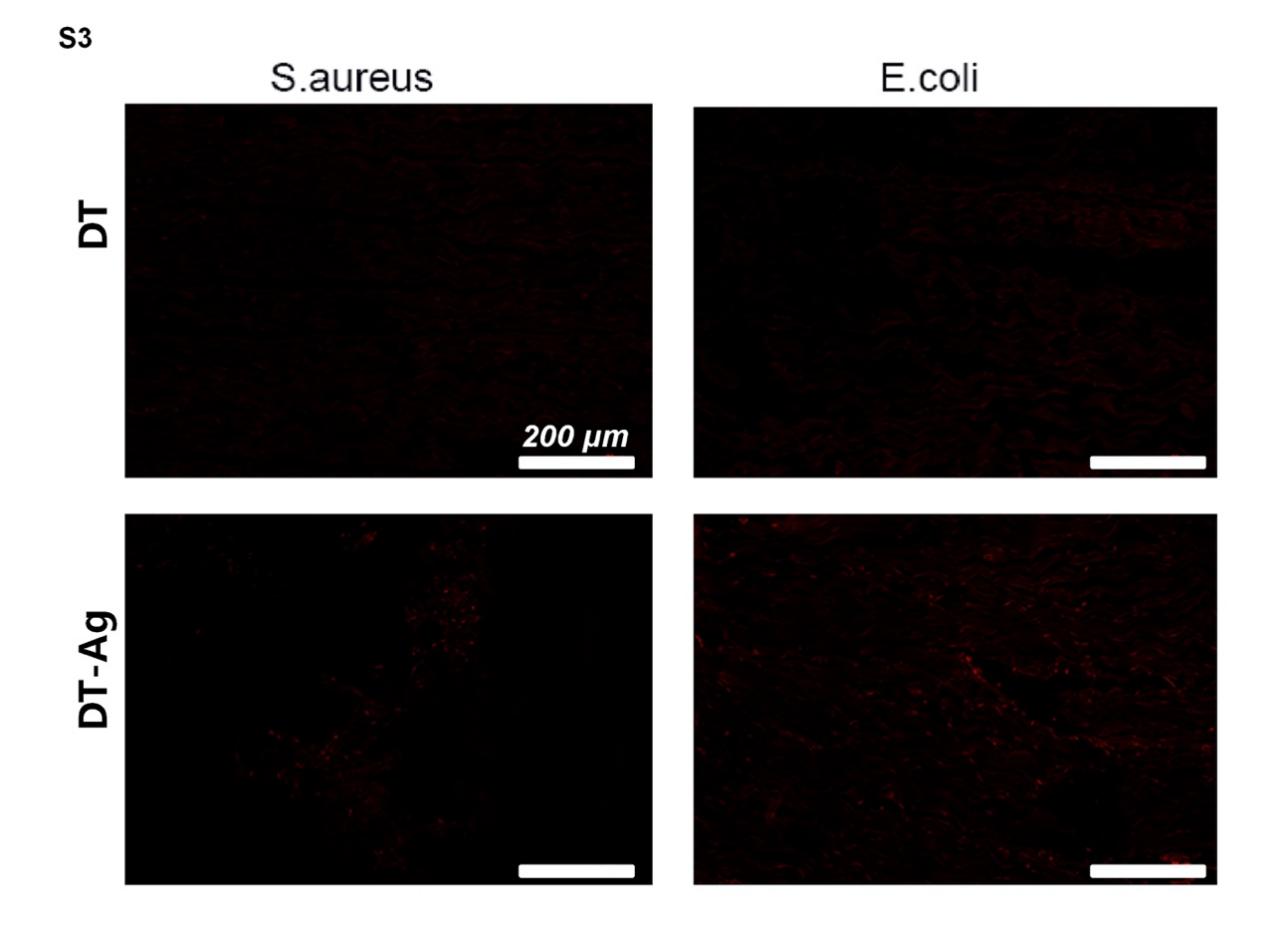


**Fig. S3** Propidium iodide staining images of *Staphylococcus aureus* and *Escherichia coli* on DT and DT-Ag surfaces
